# Applicability of Compost and Mineral Materials for Reducing the Effect of Diesel Oil on Trace Element Content in Soil

**DOI:** 10.3390/ma16103655

**Published:** 2023-05-11

**Authors:** Mirosław Wyszkowski, Natalia Kordala

**Affiliations:** Department of Agricultural and Environmental Chemistry, University of Warmia and Mazury in Olsztyn, Łódzki 4 Sq., 10-727 Olsztyn, Poland

**Keywords:** diesel oil contamination, compost, bentonite, calcium oxide, trace elements, soil

## Abstract

Petroleum-derived substances have become the factor adversely affecting the soil quality and, also, crop production. However, the ability to immobilise contaminants is limited in anthropogenically altered soils. Therefore, a study was undertaken to evaluate the effects of soil contamination with diesel oil (0, 2.5, 5 and 10 cm^3^ kg^−1^) on the contents of trace elements in the soil and determine the suitability of different neutralising materials (compost, bentonite and calcium oxide) for the in situ stabilisation of soil contaminated with this petroleum derivative. In the soil contaminated with the highest dose of diesel oil (10 cm^3^ kg^−1^), a decrease in chromium, zinc and cobalt and an increase in the total nickel, iron and cadmium concentrations were found in the series without the addition of neutralising materials. Remediation with compost and mineral materials contributed to a significant reduction of nickel and iron, as well as cobalt, in soil (calcium oxide only). All materials used contributed to an increase in cadmium, chromium, manganese and copper in the soil. The above-mentioned materials (most notably calcium oxide) can be successfully used to reduce the effect of diesel oil on the contents of some trace elements in soil.

## 1. Introduction

Soil is one of the main natural resources of the Earth and plays a key role in the functioning of ecosystems [1]. Economic activities and the existence of societies depend heavily on the ecosystem performance of soil [2], representing a natural capital [3], as well as a spatial and environmental production workshop [4,5]. However, contemporary anthropogenic activity contributes to soil environment degradation not only in individual regions but also on a global scale. Soil is a limited, nonreproducible and non-renewable resource, because its degradation proceeds much faster than its formation and reclamation [6,7]. Soil contamination can elicit a persistent impact on the environment and socioeconomic conditions, as well as be extremely difficult and expensive to remove [7]. The area of arable land is limited, and the loss of productivity caused by soil degradation as a result of anthropogenic factors poses a serious threat to food security [8].

Petroleum-derived substances are the most common cause of soil environment contamination due to their commonness and wide availability [9,10]. Diesel oil pervades the soil mainly during production, transport and as a result of accidents during fuel storage [11]. In addition to alkanes, it contains polycyclic aromatic hydrocarbons, which are severely harmful to humans due to their toxic and carcinogenic properties [12]. Due to their nonpolar and hydrophobic nature, petroleum components and petrochemical products spread across the soil mainly in the form of water-immiscible liquids upon the action of gravity and capillary forces in both vertical and horizontal directions [13]. The above-mentioned migration processes of petroleum products are conducive to their rapid and uncontrolled spread, and their long-term accumulation poses a threat to the quality of surface waters and groundwaters and constitutes a secondary source of contamination [14,15]. Soil contamination with petroleum derivatives, including diesel oil, reduces soil fertility, disturbs soil ecological homeostasis [15], suppresses the activity and reduces the biodiversity of microorganisms [16], impairs biochemical cycles and adversely affects plant production [17,18]. When diesel oil pervades the soil, it adversely affects air and water management and reduces the capacity of the soil sorption complex [19,20], as well as distorts the proportions of carbon to nitrogen and phosphorus [20] and modifies the contents of trace elements in plant organs [21]. Soil contamination with diesel fuel can also increase the contents of many trace elements in soil, such as cadmium, lead, copper, nickel and manganese [20,22,23]. However, its effect on trace element contents in soil depends on the level of diesel pollution, as well as the soil properties [23]. Numerous studies have confirmed the obstructive effect of diesel oil contamination on plant physiology and morphology. It has been proven to reduce seed germination [24,25] and nutrient transfer [26] and to induce oxidative stress [27]. In addition, it has been demonstrated to impair the metabolic activity of plants, to minimise leaf growth and cause leaf deformation, leading to the necrosis of plant tissues and cells [28], and ultimately leads to a reduction in plant yield [29,30].

For these reasons, the effective removal of diesel oil from the environment is of utmost importance. Searching for effective methods of reclamation and undertaking action to develop contaminated areas comes from the common concern for sustainable development. There are many remediation methods for an environment contaminated with both organic and inorganic compounds. Some of these technologies, such as soil vapor extraction, soil incineration or soil washing, are highly energy-intensive and costly [31]. Their implementation often requires the use of specially dedicated installations, and despite the removal of harmful substances, this often leads to soil quality deterioration [32]. For this reason, in situ stabilisation is a widely explored method for the reclamation of chemically degraded areas. This technique is much more effective in terms of protecting the soil environment, with low financial outlays [33,34]. In addition, in situ immobilisation techniques cause no damage to the structure and biological activity of the soil [18,35] and prevent secondary contamination [33].

The materials used in these processes enable the effective immobilisation of contaminants in the soil matrix, limiting their vertical migration or runoff along the surface with rainwater [35]; the most common materials used to this end so far include lime [36,37], organic matter (e.g., in the form of compost) [32,38] and other sorbents [39,40,41]. Treatments with these materials are aimed at increasing the biodegradation potential of soil by increasing its pH or its sorption capacity [42,43]. The sorption capacity largely depends on the amount of organic matter. The greater its content, the greater the sorption capacity of the soil and, thus, its ability to bind contaminants and detoxify the environment [44,45]. In soils with a high content of organic matter or clay minerals, petroleum compounds are readily absorbed and can be more easily degraded, converted and taken up by various microorganisms [46]. According to the findings from some experiments, compost application not only affects the sorption potential of soil but also provides nutrients to autochthonous microorganisms, enabling them to effectively degrade hydrocarbon contaminants [47,48]. Good results in reducing the impact of diesel pollution in soil were also achieved for trace elements in soil. Both mineral and organic amendments had positive limiting effects on trace element contents in soil [49,50]. However, it should be noted that the effects of mineral amendments on soil trace element contents are usually greater than those of organic amendments [50]. The application of these materials has a positive effect on soil properties by reducing soil acidity and reducing the contents of plant-available forms of trace elements in soil.

The development of industry entails transformations of the natural environment. Therefore, a study was undertaken to (1) assess the effect of soil contamination with diesel oil on the contents of trace elements in soil and (2) determine the suitability of different neutralising materials (compost, bentonite and calcium oxide) for the in situ stabilisation of soil contaminated with this petroleum derivative.

## 2. Material and Methods

### 2.1. Vegetation Research Methodology

The soil used to establish a vegetative pot experiment was collected from the top layer of 0–25 cm of proper brown soil with the granulometric composition of light loamy sand [51]. It was a slightly acidic soil with pH_KCl_—5.54 (±0.11), hydrolytic acidity (HAC)—23.2 (±0.5) mmol(+) kg^−1^, total exchangeable bases (TEB)—107.0 (±2.7) mmol(+) kg^−1^, cation exchange capacity (CEC)—130.2 (±3.2) mmol(+) kg^−1^ and base saturation (BS)—82.2 (±2.1)%. Its chemical composition was as follows: content of C_org_—6.34 (±0.09) g kg^−1^ and contents of available phosphorus—29.32 (±0.35) mg kg^−1^, potassium—51.78 (±1.09) mg kg^−1^ and magnesium—62.48 (±1.12) mg kg^−1^. The research was carried out in a greenhouse belonging to the University of Warmia and Mazury in Olsztyn (North-eastern Poland). The soil was contaminated with diesel oil in doses of 0, 2.5, 5 and 10 cm^3^ kg^−1^. Analyses were carried out in four series: without amendments and with the addition of compost, bentonite and calcium oxide at the following doses: compost—3%, bentonite—2% of the soil weight in the pot and calcium oxide (50% CaO) in a dose corresponding to one full hydrolytic acidity. At the time of establishing the experiment, the soil (9 kg) was thoroughly mixed with these materials and diesel oil, as well as with macronutrients and micronutrients, which doses were the same in all pots: 25 mg of nitrogen, 30 mg of phosphorus, 70 mg of potassium, 50 mg of magnesium, 5 mg of manganese, 5 mg of molybdenum and 0.33 mg of boron per kg of soil. Soil prepared in this way was placed in polyethylene pots. The contents of the trace elements in the soil and the materials used (compost, bentonite and calcium oxide) were determined before the experiment was established. The results of these assays were provided in our previous work [52]. The test plant was maize (*Zea mays* L.) of the Scandia cultivar; its density was 8 plants per pot. Maize was chosen for the experiment because of its large biomass, which is important in the remediation of contaminated soils, as well as its agricultural and economic importance. During plant growth and development, soil moisture was maintained at 60% of the maximum water capacity. The experiment was carried out with 4 repetitions. After maize was harvested at the tasselling stage BBCH 59 (Biologische Bundesanstalt, Bundessortenamt and Chemical Scale), 66 days after sowing, soil samples were collected for laboratory analyses.

### 2.2. Methodology of Laboratory Analyses

The collected samples of the soil material were dried and sieved through a screen. Soil samples prepared in this way were mineralised in a mixture of concentrated hydrochloric acid (HCl p.a.—1.18 g cm^−3^) and nitric acid (HNO_3_ p.a.—1.40 g cm^−3^) in a MARS 6 microwave oven (CEM Corporation, Matthews, NC, USA) using the US-EPA3051 methodology [53]. Then, the total contents of cadmium, lead, chromium, nickel, zinc, copper, manganese, iron and cobalt were determined by atomic absorption spectrometry (AAS) using a SpectrAA 240FS spectrophotometer (Varian Inc., Mulgrave, VIC, Australia) [54]. Certified Analytical Reference Material Soil S-1 (AGH University of Science and Technology, Krakow, Poland) and Fluka standard materials (51994 for Cd, 16595 for Pb, 02733 for Cr, 42242 for Ni, 188227 for Zn, 38996 for Cu, 63534 for Mn, 16596 for Fe and 119785.0100 for Co) were used to ensure high precision of the determinations.

Additionally, analyses of the soil properties were carried out before the experiment was established, which included determinations of the pH (pH_KCl_) of the soil with the potentiometric method [55], the hydrolytic acidity (HAC) and the total exchangeable bases (TEB) with the Kappen method [56], the contents of the available phosphorus and potassium with the Egner-Riehm method [57] and the available magnesium with the Schachtschabel method [58]. After determining the hydrolytic acidity and the sum of the alkaline cations, the total cation exchange capacity of the soil (CEC) and base saturation (BS) were calculated according to the formulas CEC = TEB + HAC and BS = TEB·CEC^−1^· 100 [54]. Laboratory analyses of the soil were carried out with 3 replicates.

### 2.3. Methodology of Statistical Analysis

The significance of the effects of the studied factors was calculated with Statistica 13 software [59] using two-way ANOVA, Tukey’s HSD test, a principal component analysis (PCA) and percentages of the observed variations, as well as Pearson’s simple correlation coefficients. Statistical calculations were performed at a significance level of ** *p* ≤ 0.01, and the correlation coefficients were additionally computed at * *p* ≤ 0.05.

## 3. Results

Soil contamination with diesel oil, as well as the application of compost, bentonite and calcium oxide, the suitability of which as neutralising materials was tested in this experiment, significantly affected the contents of trace elements in the soil (Table 1, Table 2 and Table 3).

In the series without the addition of remediating materials, increasing doses of diesel oil resulted in a statistically significant reduction in the contents of chromium (50.0%), zinc (33.5%) and cobalt (59.8%) in the soil compared to the control (uncontaminated) soil (Table 1, Table 2 and Table 3). In the same experimental series, the contents of nickel and iron were positively correlated with soil contamination using the petroleum-derived substance. The highest tested dose of diesel oil (10 cm^3^ kg^−1^) increased the contents of these elements in the soil increased by 31.2% and 24.4%, respectively. In contrast, no significant effect was noted for lead, manganese and copper. In the case of cadmium, the effect of soil contamination with diesel oil was inexplicit, i.e., doses up to 5 cm^3^ kg^−1^ caused a slight reduction in its content in the soil, while the highest dose increased its content by 18.9% compared to the control object.

The materials (compost, bentonite and calcium oxide) used to remediate soil contaminated with diesel oil exerted various effects on the contents of trace elements in the soil (Table 1, Table 2 and Table 3), depending on the extent of soil contamination and element type. All neutralising materials significantly reduced the contents of nickel and iron in the soil. Calcium oxide was found to most effectively reduce nickel accumulation. In the series with soil contaminated with diesel oil at a dose of 10 cm^3^ kg, the application of calcium oxide reduced the nickel content by 68.7% compared to the sample not amended with remediating agents. In the same experimental series, bentonite reduced the nickel content of the soil by 65.3% and compost by 55.6%. The materials used also reduced the iron content in the soil by 39.4% (bentonite), 27.0% (compost) and 20.1% (calcium oxide) compared to the control (without the remediating materials). In addition, the introduction of calcium oxide to the soil contaminated with the highest diesel oil dose tested reduced the cobalt accumulation in the soil by 39.0% compared to the soil without its application.

The effect of compost on the contents of trace elements in soil contaminated with diesel oil, although positive, was weaker than that of bentonite and calcium oxide (Table 1, Table 2 and Table 3). Its application to the soil contaminated with the highest diesel oil dose tested promoted the accumulation of all analysed trace elements, except for nickel and iron. The greatest changes were noted for chromium and lead, which contents in the soil increased seven-fold and two-fold, respectively, compared to the control variant (without the addition of a neutralising material). No significant effect was noted for cobalt. Interestingly, the application of compost to the uncontaminated soil contributed to reduced contents of nickel and iron while increasing those of cadmium, lead, copper and zinc.

The application of bentonite to the soil contaminated with the highest tested dose of diesel oil contributed to an increase in the contents of cadmium, chromium, manganese and copper in the soil, with the greatest changes observed for the contents of chromium (six-fold increase) and cadmium (two-fold increase) compared to the soil not amended with this neutralising agent (Table 1, Table 2 and Table 3). No significant effect was shown for lead, zinc and cobalt. On the other hand, bentonite introduced into the uncontaminated soil caused a statistically significant decrease only in the content of chromium (45%) and an increase in the lead content (two-fold increase). Changes in the contents of the other elements tested were statistically insignificant.

Calcium oxide application to the soil contaminated with a diesel oil dose of 10 cm^3^ kg^−1^ promoted the accumulation of cadmium (21.4%), manganese (10.3%), copper (85.5%), chromium (638%) and lead (91.2%) in the soil compared to the soil sample not remediated with this agent. This effect was not observed for zinc. The liming of the soil not contaminated with diesel oil caused no statistically significant increase in the contents of most of the analysed elements, except for nickel, which average content was higher by 43.7% than in the control soil.

**Figure 1:**
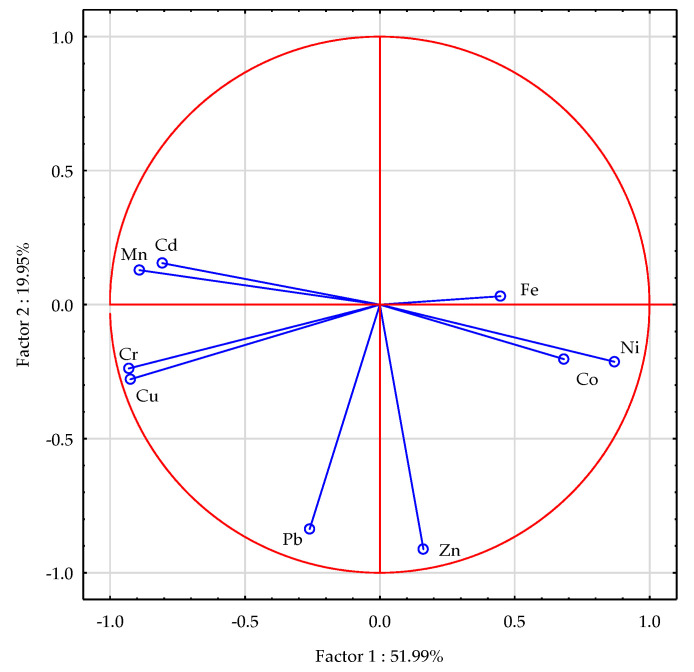
Contents of trace elements in the soil illustrated with the PCA method. Key: vectors represent analysed variables (contents of Cd, Pb, Cr, Ni, Zn, Cu, Mn, Fe and Co).

**Figure 2:**
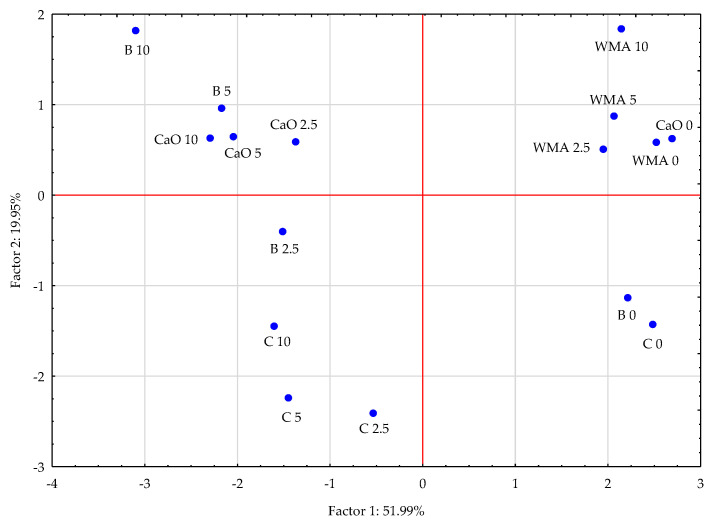
Effects of amendments in the contents of trace elements in the soil illustrated with the PCA method. Key: points show the samples with elements (WMA—without material amendments, C—compost, B—bentonite and CaO—calcium oxide); 0—0 cm^3^ (control), 2.5—2.5 cm^3^, 5—5 cm^3^ and 10—10 cm^3^ diesel oil per kg of soil.

The Principal Component Analysis demonstrated that the portion of the studied factors accounted for 71.94% of the total correlation of the data set (Figure 1). Comparing the lengths of the vectors characterising the contents of the analysed trace elements in the soil, it was found that the cobalt, cadmium and, especially, iron vectors were shorter than the others, which indicates their smaller contributions to the correlation of the data set. Positive and negative correlations were found between the contents of individual trace elements in the soil. The contents of copper and chromium in the soil were positively correlated with the contents of cadmium and manganese, that of lead with zinc and, to a lesser extent, that of nickel with cobalt and iron. In contrast, a negative correlation was found between the contents of nickel and cobalt in the soil and the contents of copper, chromium, cadmium and manganese. Soil amendment with the neutralising materials usually reduced the contents of the analysed trace elements in the soil (Figure 2), with calcium oxide and bentonite observed to exert the strongest effects.

**Figure 3:**
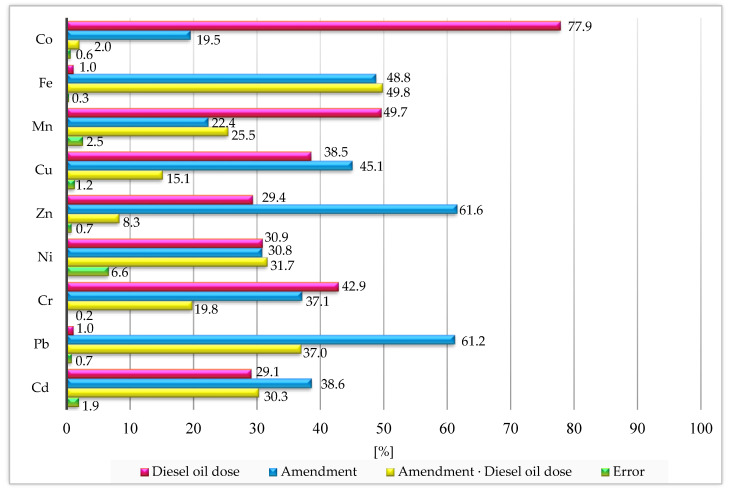
Percent contributions of variable factors according to the contents of the trace elements in the soil.

The cumulative effect of the studied factors (soil contamination with diesel oil and the application of various materials to the soil) on the contents of the trace elements in the soil based on the percentages of the observed variabilities is shown in Figure 3. Soil contamination with diesel oil had a stronger effect than neutralising agents on the contents of cobalt (77.9%), manganese (49.7%) and chromium (42.9%). In turn, the remediating materials introduced into the soil had a stronger effect than diesel oil on the contents of copper (45.1%), zinc (61.6%), lead (61.2%), iron (48.8%) and cadmium (38.6%). Soil contamination with diesel oil also had a significant effect on the nickel content (30.9%).

## 4. Discussion

In the present study, increasing doses of diesel oil contributed to a significant reduction in the contents of chromium, zinc and cobalt in the soil and promoted the accumulation of nickel and iron compared to the control (uncontaminated) soil. The effects noted for the other elements were not that obvious. According to Grujić et al. [60], in soil contaminated with petroleum derivatives, nickel is bound with manganese oxides, which can be easily reduced, becoming the predominating source of this element in the topsoil. Wyszkowski and Sivitskaya [61] demonstrated a similar effect of soil contamination with fuel oil, which has similar properties to diesel oil, on the contents of chromium, lead and manganese in soil. Soil contamination with diesel oil has been found to contribute to increased contents of trace elements, including cadmium, lead, copper or manganese [22,23]. This observation was confirmed in the research conducted by Gospodarek et al. [20] who noted an increased accumulation of manganese, lead, nickel and cadmium in soil contaminated with diesel oil at a dose of 6 g kg^−1^ soil d.m. Additionally, the findings reported by Agbogidi et al. [62] and Wyszkowski and Kordala [50] confirmed the stimulating effects of petroleum-derived contaminants on the contents of trace elements in soil.

The influence of the long-term exploitation of crude oil (since 1970) and the spill of petroleum hydrocarbons on the contents of trace elements in the soil of the Yellow River Delta in Shandong Province in China was assessed by Fu et al. [63]. They collected 22 surface soil samples (0–20 cm deep) from the Shengli oil field area and noted increased levels of zinc, nickel and cadmium. The contents of chromium, copper, zinc and vanadium in the soil were also increased but did not exceed the national environmental standards. Soil contamination with petroleum derivatives modifies its physicochemical properties. It contributes to increased soil acidification, expressed by a pH value decrease [64], while reducing the sum of exchangeable base cations, the total cation exchange capacity and the degree of soil saturation with base cations [65]. A decrease in the pH value of soil contaminated with diesel oil was also demonstrated by Bona et al. [66], Rusin et al. [67] and Jabbarov et al. [68]. Under these conditions, the mobility and availability of trace elements increase as a result of increasing the solubility of the chemical bonds of these elements and their reduced adsorption on soil colloids [69]. In addition, petroleum substances pervading soil reduce its redox potential (Arroyo et al. [70]), which results in an increase in the contents of soluble forms of trace elements in the soil environment. These changes in soil properties in response to its contamination with petroleum derivatives may explain the increased accumulation of nickel and iron noted in this study.

Calcium oxide and bentonite used in the present research had a stronger effect on reducing the contents of trace elements in the soil exposed to the pressure of diesel oil than compost. The introduction of bentonite into the soil contributed to the reduced accumulation of nickel and iron. This effect could be due to a large specific surface area of the sorbent (80–430 m^2^ g^−1^) [49,71] and its high cation exchange capacity [72]. In addition, clay rocks such as bentonite are characterised by a large pore volume, negative surface charge and a hydrophilic surface [73], which makes them effective absorbers of inorganic contaminants. The immobilisation of trace elements by clay materials occurs as a result of (1) the complexation of the surface at the edge sites, (2) exchange of cations [74] or (3) intercalation, i.e., the substitution of a cation in the internal structure of the mineral [75]. Bentonite suitability for the remediation of soil contaminated with trace elements was demonstrated by Kumararaja et al. [76]. Its 2.5% addition resulted in a statistically significant decrease in the labile fraction of zinc (18.0%), copper (28.0%) and nickel (35.0%) in the soil compared to the control series (without the bentonite addition). Usman et al. [77] assessed the effects of the addition of three clay minerals (Na-bentonite, Ca-bentonite and zeolite) on the contents of trace elements and the biochemical parameters of soil from a sewage sludge storage area. They observed that Na-bentonite and Ca-bentonite had stronger effects than zeolite on reducing the contents of water-extractable trace elements in the soil. They reduced the content of zinc in the soil by 51.0% and 48.0%, cadmium by 75.0% and 68.0%, copper by 57.0% and 69.0% and nickel by 59.0% and 29.0%, respectively, compared to the control soil. According to Usman et al. [77], this was due to the higher proportion of montmorillonite in bentonite (62–70%) than in zeolite (10%). In addition, both bentonites had a positive effect on the mineralisation process, metabolic quotient (qCO_2_; marker of environmental stress of the microbial population) and the release of inorganic nitrogen.

Most clay materials, including bentonite, sepiolite and palygorskite, are alkali minerals. Their addition to soil usually increases its pH and has a positive effect on the remediation of contaminated acidic soils. However, as reported by Sun et al. [78], bentonite also effectively induces the immobilisation of trace elements in alkaline soil. These authors demonstrated that the application of this mineral to alkaline soil (pH 8.2) contaminated with trace elements resulted in a decrease content of the exchangeable fraction of cadmium (from 10.1% to 42.5%) and lead (from 20.3% to 49.3%). Bentonite, especially when chemically modified, can be successfully deployed to remove hydrocarbons from the environment and remediate soils contaminated with petroleum substances. This was confirmed in the research conducted by Chikwe et al. [79], who, by subjecting bentonite to organophilisation with dodecyltrimethylammonium bromide (DTAB), increased its adsorption capacity and affinity to organic solvents, including diesel oil.

In the present study, calcium oxide application to the soil contaminated with the highest tested dose of diesel oil (10 cm^3^ kg^−1^) contributed to a statistically significant reduction in the contents of nickel, iron and cobalt. These results are, in part, consistent with the findings from our previous research (Wyszkowski and Kordala [50]), which showed that the application of calcium oxide to soil contaminated with gasoline resulted in a decrease in the contents of nickel (11%), cadmium (26.0%), chromium (55.0%) and cobalt (33.0%) compared to the series without the addition of a neutralising material. Xu et al. [80] proved that the addition of lime (0.2%) could significantly reduce the exchangeable fraction of copper and cadmium in soil by 81.1–85.6% and 46.3–55.9%, respectively. Additionally, Vondráčková et al. [81] and Kumarpandit et al. [82] confirmed the effectiveness of the liming process in the in situ stabilisation of contaminated soils. In turn, Kumarpandit et al. [82] reported a 28.4% reduction in the cadmium content in soil after 60 days of application of calcium carbonate (30 g per 15 kg of soil) compared to that found in non-limed soil. In their opinion, the introduction of alkaline compounds into the soil promoted the preferential sorption of trace elements by various soil colloids. The differences observed between the present research results and those reported by the above-mentioned authors may be due to different types of neutralising materials used in the liming process and the doses, as well as to the types of soil and their granulometric compositions. The immobilisation of trace elements in contaminated soil subjected to liming is mainly caused by an increase in soil pH [83,84], as well as an increase in the negative charge, precipitation of elements in the form of hydroxides and sequestration due to enhanced microbial activity [85]. The limited mobility of trace elements as a result of soil pH regulation was noted by, among others, Liu et al. [86] and Jalali and Najafi [87].

Some of the most important soil factors that determine the solubility and availability of trace elements include soil pH and the organic matter content [83,88]. The contents of trace elements and their potential availability to plants can be modified by influencing the sorption properties of soil through the addition of neutralising materials. The in situ stabilisation of contaminated soils is a cheap and technologically simple technique. Its unquestionable advantage is the high availability of immobilising agents and their long-lasting effects on soil [89]. By preventing the biomagnification of trace elements in the ecosystem, this process of remediating contaminated soils significantly minimises the threat posed to the natural environment and to human health and life [83].

## 5. Conclusions

Soil contamination with diesel oil in the series without neutralising materials caused a significant increase in the contents of nickel and iron and a decrease in the contents of chromium, zinc and cobalt in the soil compared to the uncontaminated soil. In the cases of the other trace elements tested, there were no significant changes, or the effect of diesel oil was ambiguous.

Additionally, the remediating materials had a significant effect on the contents of the trace elements in the soil. Their application resulted in a significant reduction in the average total contents of iron and nickel, as well as cobalt (only in the series with calcium oxide), while promoting the accumulation of chromium, manganese, copper and cadmium in the soil. Among the neutralising materials tested, bentonite and calcium oxide proved best in reducing the contents of the trace elements in the soil contaminated with diesel oil. The above-mentioned materials (most notably calcium oxide) can be successfully used to reduce the impact of diesel on the contents of some trace elements in soils.

## Figures and Tables

**Table 1:** The contents of cadmium, lead and chromium in the soil (mg kg^−1^ d.m.).

Material	Diesel Oil Dose (cm^3^ kg^−1^ d.m. of Soil)				Average	*r*
	0	2.5	5	10		
Cadmium (Cd)						
Without amendments	0.212 *^ab^*	0.198 *^ab^*	0.198 *^ab^*	0.252 *^a–c^*	0.215 *^B^*	0.741 **
Compost	0.265 *^a–c^*	0.360 *^cd^*	0.216 *^ab^*	0.360 *^cd^*	0.300 *^A^*	0.354
Bentonite	0.194 *^ab^*	0.540 *^e^*	0.522 *^e^*	0.504 *^e^*	0.440 *^C^*	0.616 *
CaO	0.141 *^a^*	0.432 *^de^*	0.450 *^de^*	0.306 *^bc^*	0.332 *^A^*	0.315
Average	0.203 *^B^*	0.383 *^A^*	0.347 *^A^*	0.356 *^A^*	0.322	0.591 *
Lead (Pb)						
Without amendments	15.63 *^a^*	19.70 *^a^*	17.70 *^a^*	16.65 *^a^*	17.42 *^A^*	−0.027
Compost	33.00 *^cd^*	33.28 *^cd^*	32.93 *^cd^*	35.13 *^d^*	33.59 *^D^*	0.856 **
Bentonite	33.74 *^cd^*	29.40 *^c^*	19.35 *^a^*	17.90 *^a^*	25.10 *^C^*	−0.912 **
CaO	15.63 *^a^*	19.55 *^a^*	24.45 *^b^*	31.83 *^cd^*	22.87 *^B^*	0.998 **
Average	24.50 *^AB^*	25.48 *^A^*	23.61 *^B^*	25.38 *^A^*	24.74	0.226
Chromium (Cr)						
Without amendments	12.74 *^b^*	12.09 *^b^*	9.62 *^ab^*	6.37 *^a^*	10.21 *^B^*	−0.988 **
Compost	6.75 *^a^*	48.10 *^g^*	48.75 *^g^*	44.20 *^e–g^*	36.95 *^A^*	0.615 *
Bentonite	7.01 *^a^*	34.32 *^c^*	39.39 *^d^*	39.65 *^de^*	30.09 *^C^*	0.765 **
CaO	6.63 *^a^*	43.29 *^d–f^*	47.19 *^fg^*	47.06 *^fg^*	36.04 *^A^*	0.730 **
Average	8.28 *^C^*	34.45 *^AB^*	36.24 *^B^*	34.32 *^A^*	28.32	0.670 *

*r*—correlation coefficient, ** *p* ≤ 0.01 and * *p* ≤ 0.05. Values with different letters are significantly different at *p* ≤ 0.01: *^A–C^* for the petrol dose, *^A–D^* for the material amendments and *^a–g^* for the interactions between the petrol dose and the material amendments (ANOVA and Tukey’s HSD test).

**Table 2:** The contents of nickel, zinc and copper in the soil (mg kg^−1^ d.m.).

Material	Diesel Oil Dose (cm^3^ kg^−1^ d.m. of Soil)				Average	*r*
	0	2.5	5	10		
Nickel (Ni)						
Without amendments	14.27 *^a^^–^^f^*	15.08 *^b^^–^^f^*	17.94 *^d^^–^^f^*	18.72 *^ef^*	16.50 *^B^*	0.933 **
Compost	21.02 *^f^*	16.90 *^c^^–^^f^*	14.17 *^a^^–^^f^*	8.32 *^a^^–^^c^*	15.10 *^B^*	−0.997 **
Bentonite	14.78 *^a^^–^^f^*	8.97 *^a^^–^^d^*	6.76 *^ab^*	6.50 *^ab^*	9.25 *^A^*	−0.826 **
CaO	20.51 *^ef^*	11.44 *^a^^–^^e^*	8.45 *^a^^–^^c^*	5.85 *^a^*	11.56 *^A^*	−0.892 **
Average	17.65 *^B^*	13.10 *^A^*	11.83 *^A^*	9.85 *^A^*	13.11	−0.919 **
Zinc (Zn)						
Without amendments	28.85 *^c^*	28.38 *^c^*	26.96 *^bc^*	19.19 *^a^*	25.85 *^B^*	−0.946 **
Compost	34.20 *^d^*	40.78 *^e^*	40.68 *^e^*	33.85 *^d^*	37.38 *^C^*	−0.207
Bentonite	30.35 *^cd^*	29.61 *^c^*	24.33 *^b^*	18.27 *^a^*	25.64 *^AB^*	−0.981 **
CaO	28.07 *^bc^*	27.50 *^bc^*	24.36 *^b^*	17.40 *^a^*	24.33 *^A^*	−0.976 **
Average	30.37 *^B^*	31.57 *^C^*	29.08 *^AB^*	22.18 *^A^*	28.30	−0.912 **
Copper (Cu)						
Without amendments	2.524 *^a^*	2.700 *^a^*	2.725 *^a^*	2.750 *^a^*	2.675 *^C^*	0.812 **
Compost	2.548 *^a^*	5.650 *^d^*	5.450 *^cd^*	5.000 *^b^^–^^d^*	4.662 *^B^*	0.533 *
Bentonite	3.210 *^a^*	4.500 *^b^*	5.150 *^b^^–^^d^*	5.300 *^b^^–^^d^*	4.540 *^AB^*	0.865 **
CaO	2.744 *^a^*	4.500 *^b^*	4.550 *^bc^*	5.100 *^b^^–^^d^*	4.224 *^A^*	0.846 **
Average	2.757 *^B^*	4.338 *^A^*	4.469 *^A^*	4.538 *^A^*	4.025	0.746 **

*r*—correlation coefficient, ** *p* ≤ 0.01 and * *p* ≤ 0.05. Values with different letters are significantly different at *p* ≤ 0.01: *^A–C^* for the petrol dose, *^A–C^* for the material amendments and *^a–f^* for the interactions between the petrol dose and the material amendments (ANOVA and Tukey’s HSD test).

**Table 3:** The contents of manganese, iron and cobalt in the soil (mg kg^−1^ d.m.).

Material	Diesel Oil Dose (cm^3^ kg^−1^ d.m. of Soil)				Average	*r*
	0	2.5	5	10		
Manganese (Mn)						
Without amendments	266.3 *^a^*	272.1 *^a^^–^^c^*	276.0 *^a^^–^^c^*	271.4 *^a^^–^^c^*	271.5 *^B^*	0.468
Compost	266.2 *^a^*	281.6 *^a^^–^^d^*	311.4 *^fg^*	286.5 *^b^^–^^e^*	286.4 *^A^*	0.473
Bentonite	265.8 *^a^*	281.6 *^a^^–^^d^*	300.8 *^d^^–^^f^*	324.5 *^g^*	293.2 *^A^*	0.994 **
CaO	268.6 *^ab^*	290.0 *^c^^–^^e^*	302.4 *^ef^*	299.4 *^d^^–^^f^*	290.1 *^A^*	0.788 **
Average	266.7 *^B^*	281.3 *^C^*	297.7 *^A^*	295.5 *^A^*	285.3	0.836 **
Iron (Fe)						
Without amendments	8141 *^de^*	8443 *^e^*	9068 *^f^*	10,127 *^g^*	8945 *^C^*	0.995 **
Compost	7035 *^b^*	9647 *^g^*	7436 *^bc^*	7394 *^bc^*	7878 *^A^*	−0.172
Bentonite	8010 *^d^*	6138 *^a^*	7018 *^b^*	6138 *^a^*	6826 *^B^*	−0.669 *
CaO	7530 *^c^*	7490 *^c^*	8203 *^de^*	8086 *^de^*	7827 *^A^*	0.765 **
Average	7679 *^B^*	7930 *^A^*	7931 *^A^*	7936 *^A^*	7869	0.699 **
Cobalt (Co)						
Without amendments	3.058 *^h^*	1.980 *^fg^*	1.530 *^c^^–^^e^*	1.230 *^bc^*	1.950 *^A^*	−0.899 **
Compost	2.764 *^h^*	1.500 *^c^^–^^e^*	1.350 *^bc^*	1.320 *^bc^*	1.734 *^C^*	−0.749 **
Bentonite	3.028 *^h^*	1.800 *^e^^–^^g^*	1.770 *^d^^–^^f^*	1.440 *^cd^*	2.010 *^A^*	−0.829 **
CaO	2.146 *^g^*	1.050 *^ab^*	1.020 *^ab^*	0.750 *^a^*	1.242 *^B^*	−0.821 **
Average	2.749 *^D^*	1.583 *^C^*	1.418 *^B^*	1.185 *^A^*	1.734	−0.834 **

*r*—correlation coefficient, ** *p* ≤ 0.01 and * *p* ≤ 0.05. Values with different letters are significantly different at *p* ≤ 0.01: *^A–D^* for the petrol dose, *^A–D^* for the material amendments and *^a–h^* for the interactions between the petrol dose and the material amendments (ANOVA and Tukey’s HSD test).

## Data Availability

All data are available in the manuscripts and from the authors.
